# Linear and Nonlinear Directed Connectivity Analysis of the Cardio-Respiratory System in Type 1 Diabetes

**DOI:** 10.3389/fnetp.2022.840829

**Published:** 2022-03-08

**Authors:** Michele Sorelli, T. Noah Hutson, Leonidas Iasemidis, Leonardo Bocchi

**Affiliations:** ^1^ European Laboratory for Non-Linear Spectroscopy, University of Florence, Florence, Italy; ^2^ Department of Physics and Astronomy, University of Florence, Florence, Italy; ^3^ Department of Translational Neuroscience, Barrow Neurological Institute, Phoenix, AZ, United States; ^4^ Department of Information Engineering, University of Florence, Florence, Italy

**Keywords:** directional connectivity, type 1 diabetes (T1D), dynamical Bayesian inference, directed transfer function (DTF), cardio-respiratory system

## Abstract

In this study, we explored the possibility of developing non-invasive biomarkers for patients with type 1 diabetes (T1D) by quantifying the directional couplings between the cardiac, vascular, and respiratory systems, treating them as interconnected nodes in a network configuration. Towards this goal, we employed a linear directional connectivity measure, the directed transfer function (DTF), estimated by a linear multivariate autoregressive modelling of ECG, respiratory and skin perfusion signals, and a nonlinear method, the dynamical Bayesian inference (DBI) analysis of bivariate phase interactions. The physiological data were recorded concurrently for a relatively short time period (5 min) from 10 healthy control subjects and 10 T1D patients. We found that, in both control and T1D subjects, breathing had greater influence on the heart and perfusion with respect to the opposite coupling direction and that, by both employed methods of analysis, the causal influence of breathing on the heart was significantly decreased (*p* < 0.05) in T1D patients compared to the control group. These preliminary results, although obtained from a limited number of subjects, provide a strong indication for the usefulness of a network-based multi-modal analysis for the development of biomarkers of T1D-related complications from short-duration data, as well as their potential in the exploration of the pathophysiological mechanisms that underlie this devastating and very widespread disease.

## Introduction

Type 1 diabetes (T1D) is a chronic condition affecting roughly 5% of the world’s diabetic population (Ogurtsova et al., 2017), which is estimated to reach 642 million (95% CI: 521–829 million) by 2040 (it was 151 million in 2000 (Wild et al., 2004)) with dramatic social and financial implications. T1D is associated with pathogenetic mechanisms that lead to the apoptosis of pancreatic beta cells and, thus, to an inadequate production of the insulin hormone. There is no currently available cure for T1D, and its clinical care is focused primarily on the normalization of blood glucose levels for averting the onset of long-term complications including cardiovascular disease and renal failure. The treatment of diabetic-related chronic complications accounts for a considerable percentage [about 80% in the United Kingdom (Ogurtsova et al., 2017)] of the total medical costs of diabetes mellitus. Studies show that timing of medical intervention is key to reducing effects of comorbidities of T1D, with earlier interventions resulting in lower disease impact (Doria et al., 2012). Thus, there would be benefits to patients and healthcare systems alike from development of novel diagnostic techniques for early and non-invasive detection of T1D-related complications. Such diagnostic regimes could also have implications in outpatient monitoring and disease progression assessment.

The complex function of the cardiovascular system is realized by the synergistic activity of self-sustained cardiac, respiratory, and vascular oscillators (Ticcinelli et al., 2017), which is deemed to convey the necessary adaptability to sudden variations in the metabolic requirements of the organism or to changing environmental conditions (Penzel et al., 2017). There is a wide variety of clinically available devices for non-invasively monitoring the physiological systems that may be impacted by the progression of T1D. Such systems generate oscillatory modes that span a wide range of characteristic time scales, which can be isolated and separately characterized by means of established time-frequency representation (TFR) techniques (Clemson et al., 2016). In this regard, the wavelet transform (WT) analysis of laser Doppler flowmetry (LDF) signals of microvascular perfusion (Stefanovska et al., 1999) has contributed to the identification of myogenic (Aalkjaer et al., 2011), neurogenic (Söderström et al., 2003) and endothelial (Kvandal et al., 2006) frequency ranges in the microcirculatory vasomotion, in addition to the ones of the extrinsic cardiac and respiratory components (Stefanovska and Hozic, 2000) transmitted to the distal microvascular beds (Table 1). This, in turn, has enabled the non-invasive assessment of the underlying vasomotor mechanisms in pathological states.

**TABLE 1:** Physiological frequency ranges in microvascular perfusion signals.

Oscillation	Nominal range (Hz)
Cardiac	(0.6, 2.0)
Respiratory	(0.145, 0.6)
Myogenic	(0.052, 0.145)
Sympathetic	(0.021, 0.052)
Endothelial (NO-dependent)	(0.0095, 0.021)
Endothelial (NO-independent)	(0.005, 0.0095)

Furthermore, the wavelet cross-spectrum (Clemson et al., 2016) and the phase coherence of bivariate data, (Sheppard et al., 2012; Tankanag et al., 2014; Perrella et al., 2018), along with statistical properties translated from information theory [e.g., Granger causality (Granger, 1969) and transfer entropy (Vejmelka and Palus, 2008; Sabesan et al., 2009)], have been used to gain insights into the presence of significant relations between oscillatory sources, and to determine the existence of a mutual physiological coordination, e.g., the well-known synchronous modulation of the heartbeat period by the breathing rhythm, produced at the respiratory centers located within the medulla oblongata and pons of the brainstem (Eckberg, 2003). However, beyond the effects manifested in the oscillators’ phase dynamics, the fundamental functional mechanisms underlying these interactions can be probed via more sophisticated techniques, able to provide information about the directional strength of the coupling and hence about the causality of the interaction (Rosenblum and Pikovsky, 2001; Palus and Stefanovska, 2003; Faes et al., 2004). Since the cardiovascular system must handle time-varying conditions, the employed methods should be capable of capturing non-stationary functional couplings. The dynamical Bayesian inference (DBI) technique, more recently introduced by Stankovski et al. (2012), seeks to account for such non-stationarities. In DBI, the cardiovascular system is modelled as a network of phase oscillators coupled by time-dependent functions, which are identified dynamically through a Bayesian estimation framework within subsequent time windows of the oscillators’ phase time series. Several researchers have employed DBI to investigate potential changes in the direct and indirect coupling between the cardiac, respiratory and vasomotor activities; their studies have detected a reduction in the respiratory sinus arrhythmia with ageing (Shiogai et al., 2010; Iatsenko et al., 2013; Stankovski et al., 2014; Ticcinelli et al., 2015; Ticcinelli et al., 2017), and a weakening of the coupling between the microvascular myogenic vasomotion and the central cardiac and respiratory oscillations in the elderly population and in primary hypertension (Ticcinelli et al., 2017). Since metabolic diseases, such as obesity and diabetes, have been recognized as models of accelerated ageing, the aforementioned alterations may also be present in subjects diagnosed with T1D.

Non-stationary metrics of time-frequency activity could elucidate stochastic coupling but require an adequate number of data points over stationary windows for inferences to be statistically significant. Linearly modelling the data may provide a valuable alternative. Multivariate autoregressive (MVAR) models have been used for describing interactions between time series originating from different nodes within a network (Baccalá et al., 2007; Vlachos et al., 2017). In detail, MVAR-based parametric techniques can be utilized to elucidate inter-node connections via coherence-based measures of implicit causality. One such measure is the directed transfer function (DTF), a frequency-domain descriptor of directed network connectivity with fundamental implications from Granger causality (Baccalá et al., 2007). DTF measures cascaded direct and indirect interactions, emphasizes source-based outflow and has been employed in several neuroscience applications (Kamiński et al., 2001; Blinowska et al., 2013; Kamiński and Blinowska, 2014; Vlachos et al., 2017; Adkinson et al., 2018; Hutson et al., 2018). DTF and other MVAR-based measures of directional connectivity may also be applied to the evaluation of the directional coupling between the cardiac, respiratory, and peripheral blood flow systems. The utility of these measures in neuro-cardio-respiratory network interactions has been shown lately in animal studies of sudden unexpected death in epilepsy (SUDEP), a condition that involves potential failure of central control units of cardiac and respiratory behavior (Hutson et al., 2020).

Employing directed connectivity measures to quantify the inter-modulation of the biological oscillations originating from separate but interconnected systems could have valuable diagnostic potential for assessing the deterioration of the cardiovascular and respiratory function in prevalent high-risk conditions such as T1D. According to the results of a recent review article (Klein et al., 2010), adult subjects diagnosed with type 2 diabetes are characterized by reduced respiratory parameters, which appear to be inversely related to blood glucose levels and the time since the initial diagnosis. This review has linked chronic hyperglycemia and inflammation, autonomic neuropathy, microangiopathy of the pulmonary arterioles, and stiffening of the lung parenchyma to the possible biological mechanisms underlying the lung function impairment. This may then result in a detrimental impact on the mutual physiological coupling between the breathing and heart function. In light of the above, in the present study we employed the DBI and DTF frameworks with the aim to non-invasively detect characteristics of the potential decline of connectivity in the cardio-respiratory oscillatory network in a preliminary, relatively small group of healthy controls and patients diagnosed with T1D.

## Materials and Methods

### Experimental Setup and Subjects

**FIGURE 1:**
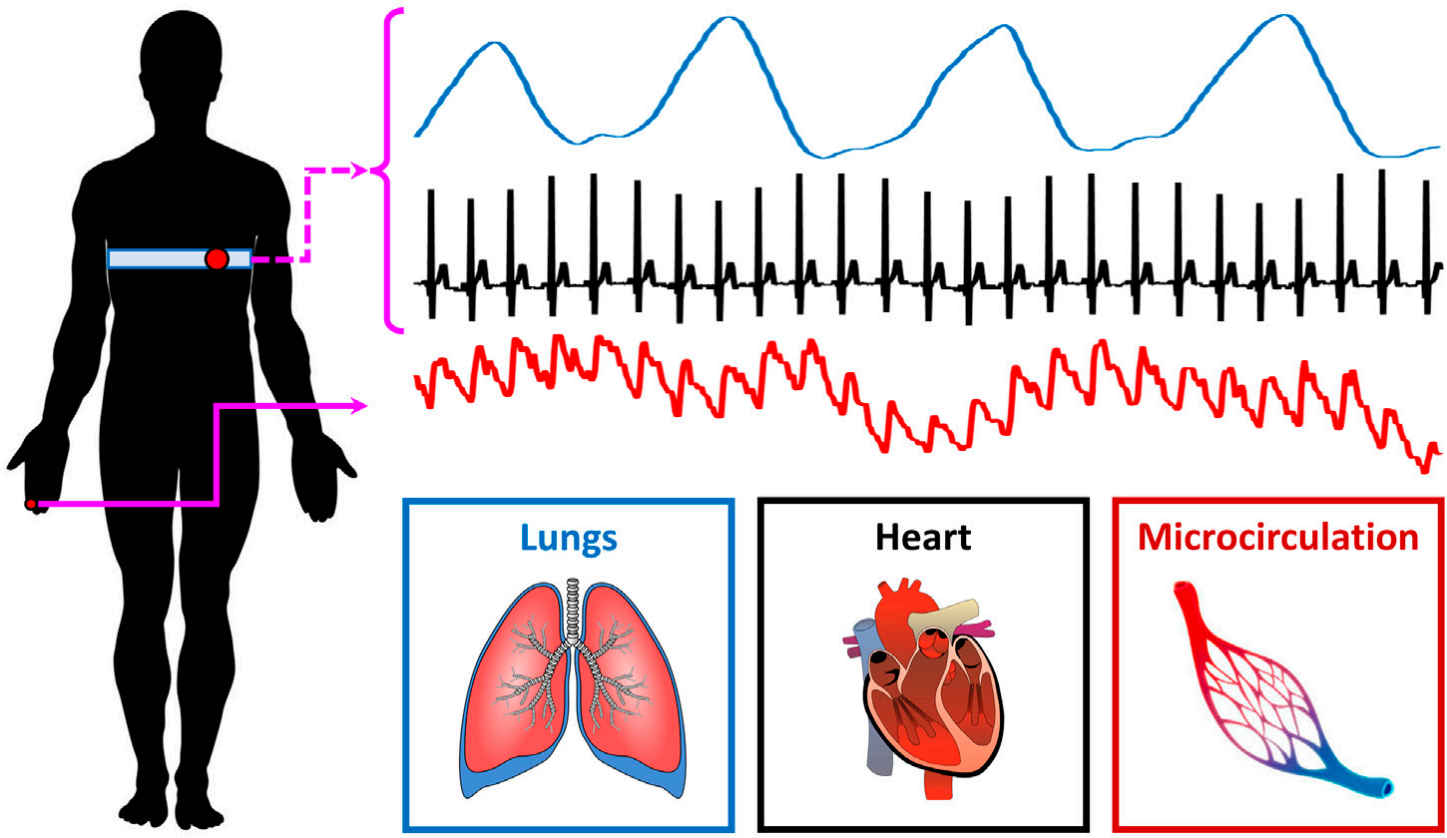
Experimental recording setup.

10 healthy controls (age: 26.7 ± 1.5 years; M/F: 7/3) and 10 T1D patients (age: 29.7 ± 13.3 years; M/F: 5/5) were recruited for the present study. Research activities were carried out in accordance with the guidelines of the Declaration of Helsinki of the World Medical Association: the included subjects received detailed information on the research protocol and its purpose and signed an informed consent form. The general characteristics of the participants are summarized in Table 2; one control subject (i.e., 10%) and four T1D subjects (40%) were smokers. ECG, breathing and microvascular perfusion signals were simultaneously recorded. Microvascular perfusion was measured on the distal phalanx of the right forefinger using a Periflux 5,000 laser Doppler flowmetry (LDF) system (Perimed AB, Sweden). The time constant of the output low-pass filter of the instrument was set to 0.03 s in order to preserve pulse waveforms. The heart and spontaneous respiratory activities were instead monitored by means of a BioHarness 3.0 wearable chest strap sensor (Zephyr Technology, United States) and transmitted to a PC *via* Bluetooth. A graphical illustration of the recording setup is shown in Figure 1.

**TABLE 2:** Study participants: general characteristics.

Characteristics		Control	T1D	*p*-value
Gender	(M/F)	7/3	5/5	0.361^α^
Age	(years)	26.7 ± 1.5	29.7 ± 13.3	1.000^β^
Smokers	(Y/N)	1/9	4/6	0.121^α^
Heart rate	(bpm)	70.7 ± 7.2	74.1 ± 9.5	0.406^β^
Breathing rate	(Hz)	0.25 ± 0.06	0.28 ± 0.03	0.149^β^
LDF cardiac power	(%)	90.6 ± 6.6	91.7 ± 7.8	0.450^β^
T1D duration	(years)	-	13.8 ± 10.0	-
HbA1c	(%)	-	7.5 ± 1.1	-

α: *via* Pearson’s $\chi^{2}$ test.

β: *via* Mann-Whitney *U* test.

**FIGURE 2:**
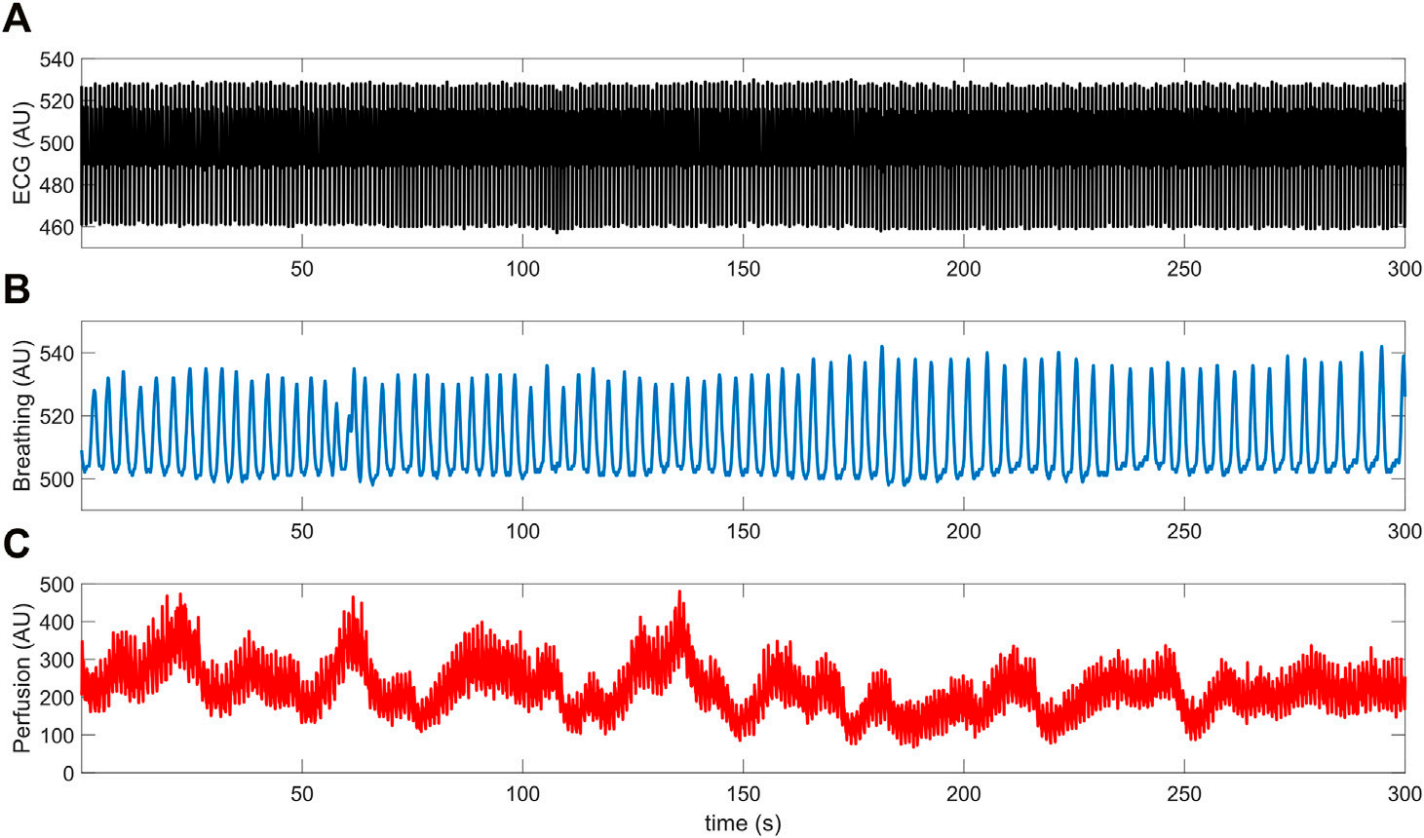
Example of **(A)** ECG, **(B)** breathing and **(C)** LDF perfusion signals recorded from a young control subject.

The above three signals were concurrently recorded and digitized at a sampling frequency of 250 Hz (being synchronized through a dedicated data acquisition software). Each recording session lasted 5 min and took place in thermally stable conditions (T ≈ 23°C) following a preliminary acclimatization time interval of 10 min. During signal acquisition, subjects were seated in a chair with back support and leaned their right forearm on a table; furthermore, they were instructed to carefully avoid abrupt movements to prevent the displacement of the LDF probe and thus the introduction of motion-related artifacts in the recorded perfusion signals. An example of the raw signals acquired from a young control individual is shown in Figure 2.

The mean breathing rate of all subjects was inside the nominal physiological range, that is (0.145, 0.6) Hz (Table 1). Furthermore, LDF perfusion signals recorded from the pathological group did not exhibit a significantly different (*p* = 0.450) power within the nominal frequency range of the cardiac rhythm (0.6, 2) Hz. However, T1D subjects included a larger proportion of smokers and were on average older than the control subjects. Nevertheless, these differences did not reach statistical significance according to Pearson’s $\chi^{2}$ and Wilcoxon rank-sum tests, respectively.

### Dynamical Bayesian Inference

The functional physiological interaction between cardiac and respiratory processes was investigated by means of the dynamical Bayesian inference (DBI) technique (Duggento et al., 2012; Stankovski et al., 2012). This method regards the cardio-respiratory system as a network of coupled self-sustained nonlinear phase oscillators and uses a Bayesian inference scheme to dynamically estimate their time-evolving coupling strength and causality (i.e., the direction of interactions). Myogenic, sympathetic, and endothelial microvascular oscillations (Table 1) were not considered in the present study, due to the insufficient duration of the recorded signals. A comprehensive description of the approach can be found elsewhere (Duggento et al., 2012; Stankovski et al., 2012; Iatsenko et al., 2013; Clemson et al., 2016; Ticcinelli et al., 2017). Briefly, in DBI, the phase dynamics of two interacting oscillatory processes $p_{1}$ and $p_{2}$ is modelled as follows:

$$\begin{array}{c} {\dot{\varphi }}_{1}\left(t\right)=\omega_{1}\left(t\right)+d_{1}\left(\varphi_{2},t\right)+k_{1}\left(\varphi_{1},\varphi_{2},t\right)+\varepsilon_{1}\left(t\right) \end{array}$$ (1)

where $\omega_{1}\left(\cdot \right)$ is the natural frequency of the first oscillator, $d_{1}\left(\cdot \right)$ and $k_{1}\left(\cdot \right)$ are the coupling functions that describe the direct and indirect driving of the second oscillator (with the acceleration/deceleration of the first oscillator’s phase $\varphi_{1}$ depending on the second’s $\varphi_{2}$ ), whereas the stochastic term, ε(⋅) , represents the noise (usually assumed to be Gaussian and white (Stankovski et al., 2012)). Since the above coupling functions are hypothesized to be 2π-periodic, the right-hand side of Eq. 1 can be decomposed into a linear combination of Fourier basis functions $\Phi_{n}=\text{exp}\left[i\left(n_{1}\varphi_{1}+n_{2}\varphi_{2}\right)\right]$ :

$$\begin{array}{c} {\dot{\varphi }}_{1}\left(t\right)=\overset{N}{\underset{n=-N}{\sum }}c_{1,n}\cdot \Phi_{1,n}\left(\varphi_{1},\varphi_{2}\right)+\varepsilon_{1}\left(t\right) \end{array}$$ (2)

where N is the order of the expansion and $\;\Phi_{i,0}=1$ (where i = 1, 2 ). In general, the DBI technique sequentially applies the Bayesian theorem to adjacent time windows of the oscillators’ instantaneous phases, $\varphi_{i}\left(t\right)$ , in order to infer the bank of time-varying parameters $c_{i,n}$ characterizing the functional interaction between the underlying physiological processes, and the noise term, $\varepsilon_{i}$ . The inferred $c_{i,n}$ values are then used to estimate a dynamic index of directional coupling strength and directionality of influence. In the present study, DBI analysis was based on the related Matlab toolbox developed by the research group on Nonlinear and Biomedical Physics at Lancaster University (http://www.physics.lancs.ac.uk/research/nbmphysics/diats/tfr/).

In detail, DBI analysis usually requires the extraction of the instantaneous frequency of the oscillations of interest, in order to track their characteristic time-dependent phase $\varphi_{i}\left(t\right)$ . In this regard, an adaptive parametric ridge reconstruction scheme (Iatsenko et al., 2016) was applied to the time-frequency representation (TFR) of the acquired signals in order to isolate the breathing and cardiac oscillatory components. In the present study, the cardiac component was isolated from both the ECG and the LDF signals of cutaneous perfusion. The adjustable parameters of the algorithm, which respectively tune the tolerance to deviations from the component’s mean rate of frequency change and mean frequency, were set to their default value of 1. The wavelet transform (WT) was adopted as TFR technique because of its logarithmic frequency resolution (Stefanovska et al., 1999); specifically, a Morlet wavelet with central frequency $f_{0}=1$ was chosen as the mother function:

$$\begin{array}{c} \gamma_{m}\left(t\right)=\frac{1}{\sqrt{2\pi }}\left(e^{i2\pi t}-e^{-\frac{{\left(2\pi \right)}^{2}}{2}}\right)e^{-t^{2}/2} \end{array}$$ (3)

**FIGURE 3:**
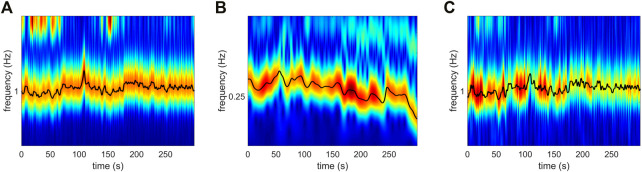
Time-frequency ridges of the **(A)** ECG, **(B)** breathing and **(C)** LDF perfusion signals shown in Figure 2. Ridges were estimated by means of the adaptive parametric approach developed in (Iatsenko et al., 2016).

Prior to the application of the WT, signals were downsampled to 50 Hz, detrended by means of a third order polynomial fit, and band-passed inside the cardiac and respiratory frequency intervals listed in Table 1, to remove the influence of components lying outside the physiological range of interest. The discretization of the frequency domain was performed with a density of 128 voices/octave, which enabled the extraction of smooth ridge curves. DBI was then applied to consecutive overlapping windows of the original time series, with an overlap factor of 50%. The window width was set so as to include approximately five cycles of the slowest oscillatory component for inference of the coupling parameters $c_{i,n}$ , as reported in (Iatsenko et al., 2015; Clemson et al., 2016). For the analysis of cardio-respiratory interactions, this resulted in the adoption of 23 overlapping windows of 25 s. Thus, the lowest frequency we could theoretically observe was 1/25 s = 0.04 Hz. The characteristic time-frequency ridges extracted from the signals in Figure 2 are shown in Figure 3.

As done by Iatsenko et al. (2013) and Ticcinelli et al. (2017), a Fourier decomposition up to the second order (i.e., *N* = 2) was chosen for the phase dynamics model expressed in Eq. 1. Moreover, the propagation constant $p_{w}$ , that weights the diffusion of information between consecutive data windows w (Stankovski et al., 2012), was set to an arbitrary value of 0.2. Iatsenko et al. (2013) have nonetheless reported that this internal parameter of the DBI algorithm does not significantly affect the outcome of the Bayesian inference. The Euclidean norm of the coupling parameters $c_{i,n}$ estimated within each data window w was finally used to quantify the overall influence (including direct and indirect couplings) of the phase of the second oscillator on the first one’s, and vice versa, yielding the following directional coupling strength signals:

$$\begin{array}{c} s_{1\to 2}\left(w\right)=\sqrt{\overset{N}{\underset{n=-N}{\sum }}{\left(c_{1,n}\left(w\right)\right)}^{2}} \end{array}$$ (4)

$$\begin{array}{c} s_{2\to 1}\left(w\right)=\sqrt{\overset{N}{\underset{n=-N}{\sum }}{\left(c_{2,n}\left(w\right)\right)}^{2}} \end{array}$$ (5)

where w indicates the dependence of the coupling coefficients on the particular time window.

Furthermore, a directionality index $d_{1,2}$ $\;\left(d_{1,2}\in \left[-1,+1\right]\right)$ was estimated from each window w , in order to quantify the dynamic asymmetry of the bi-directional interaction:

$$\begin{array}{c} d_{1,2}\left(w\right)=\frac{s_{1\to 2}\left(w\right)-s_{2\to 1}\left(w\right)}{s_{1\to 2}\left(w\right)+s_{2\to 1}\left(w\right)} \end{array}$$ (6)

This index, proposed by Rosenblum and Pikovsky (2001) has been used in the recent literature for detecting the predominant direction of influence between the cardiac and respiratory oscillators (Stankovski et al., 2012; Iatsenko, et al., 2013; Ticcinelli, et al., 2017). Namely, if $d_{1,2}\in (0,+1]$ , then the first oscillator drives the second more than the other way around; conversely, if $d_{1,2}\in [-1,0)$ , the second drives the first one. However, as reported in (Duggento et al., 2012), directional coupling strengths $s_{i\to j}\left(w\right)$ obtained via DBI represent an overall estimate of the combined phase relationships between the analyzed time series. Thus, spurious non-zero values can be inferred even when no functional interaction exists between the underlying oscillatory processes. This is why the reliability of $s_{i\to j}\left(w\right)$ should be ascertained by surrogate testing, i.e., rejecting directional coupling strengths below a specified acceptance threshold estimated from an adequately large set of surrogate interactions. In this regard, we adopted the inter-subject surrogate approach followed by Toledo et al. (2002) and Ticcinelli et al. (2017) validating our coupling strength estimates against the median value obtained from 100 unique combinations of randomly selected inter-group signals and subjects. Each of the 100 surrogate datasets was composed of mutually independent time series recorded from different individuals (e.g., ECG from control subject A, breathing from T1D patient B, LDF perfusion from control C). This technique allowed us to exclude from further consideration any directional couplings whose strength was equivalent to the one which might have arisen from chance or bias.

### Directed Transfer Function

Multivariate autoregressive (MVAR) modelling of the data within short-time segments, each data window aligned in time with concurrent ones from more than one time series, is recommended for network connectivity analysis assuming that these signals are recorded from different parts of a multi-dimensional, linear and wide-sense stationary system. For each window, the estimated array of MVAR model coefficients can then be further analyzed in the frequency domain and, depending on different types of normalization utilized, provides frequency-specific measures of directional functional connectivity between the nodes of the assumed network configuration of the system (Baccalá et al., 2007). We have successfully employed such measures in network analyses of intracranial EEG (iEEG) (Vlachos et al., 2017; Adkinson et al., 2018), and magnetoencephalographic (MEG) recordings (Krishnan et al., 2015) from patients with focal epilepsy for localization of their epileptogenic focus, as well as the assessment of the dynamics of brain’s network connections *en route* to a life-threatening neurological event, status epilepticus (T. N. Hutson et al., 2018). In the current study we fitted a MVAR model to each of 60-s consecutive non-overlapping data segments from the three recorded signals (ECG, breathing, perfusion) over 5 min. By using a 60-s time window, our frequency resolution is 1/60 s = 0.017 Hz = 0.05 Hz/3, and thus the lowest frequency we can deal with moving the analysis in the frequency domain is three times less than the 0.05 Hz, the lowest frequency in the frequency band of (0.05, 2) Hz we are interested in here. Thus, the MVAR model was of dimension D = 3 [i.e., the data to be fitted were placed in three-dimensional column vectors X(t) ], and of order M = 7 per subject. Also, the window length of 60 s (15,000 data points x three channels = 45,000 data points) is enough for a confident estimation of the 7 × 3 × 3 = 63 MVAR parameters as we are using more than 100 times as many data points as we have parameters to fit.

For each set of three 60-s running windows extracted at the same time from all three signals, the model linearly fits the data in the column vectors X(t) as follows:

$$\begin{array}{c} \begin{array}{c} X\left(t\right)=\overset{M}{\underset{\tau =1}{\sum }}A\left(\tau \right)X\left(t-\tau \right)+E\left(t\right) \end{array} \end{array}$$ (7)

where the time index t is from 1 to N, with N being the number of data points per time series within a time window (*N* = 15,000), M is the order of the model (M = 7), and τ is increasing in steps of the time delay between samples (we used τ = 1, that is, in time units, equal to the sampling period 1/(250 Hz) = 4 ms). Matrices A(τ) contain the model’s coefficients, whereas the fitting error values are the components of the vector E (in the ideal MVAR model fit, E is multivariate Gaussian white noise). The coefficients of the MVAR model were estimated via the Vieira-Morf partial correlation method. Taking the discrete Fourier Transform of both sides of Eq. 7 and rearranging, we have: $\left[I-\overset{p}{\underset{\tau =1}{\sum }}A\left(\tau \right)e^{-i2\pi f\tau }\right]\cdot X\left(f\right)=E\left(f\right)$ , where I is the unitary matrix. Then, by defining:

$$\begin{array}{c} \bar{B}\left(f\right)=\;\{\begin{array}{c} I-\overset{p}{\underset{\tau =1}{\sum }}A_{ij}\left(\tau \right)e^{-i2\pi f\tau }\;,for\;i=j\\ -\overset{p}{\underset{\tau =1}{\sum }}A_{ij}\left(\tau \right)e^{-i2\pi f\tau }\;,for\;i\neq j \end{array} \end{array}$$ (8)

where $i=\sqrt{-1}$ in the exponents of Eq. 8, the directed transfer function (DTF) can be derived by utilizing the transfer matrix, H(f) , defined as:

$$\begin{array}{c} H\left(f\right)={\bar{B}}^{-1}\left(f\right) \end{array}$$ (9)

Specifically, DTF is estimated via the following equation:

$$\begin{array}{c} DTF_{j\to i}\left(f\right)=\frac{{\left|H_{ij}\left(f\right)\right|}^{2}}{\sum_{k=1}^{D}{\left|H_{ik}\left(f\right)\right|}^{2}} \end{array}$$ (10)

The statistical significance of the DTF values of each interaction derived from each 60-s window was determined. The statistical criteria for inferring the statistical significance and confidence interval of the derived frequency-domain Granger causality-based connectivity measures are recent and have been discussed by a small number of researchers. In this study, we have followed an asymptotic analysis for evaluation of the connectivity measures from the MVAR modelling of our data (Baccalá et al., 1997; Baccalá et al., 2016). In detail, the significance of the connectivity measures $DTF_{j\to i}\left(f\right)\;$ at a specific frequency f between two nodes *i* and *j* was tested according to the following null hypothesis:

$$\begin{array}{c} \begin{array}{cc} H_{0}:{\left|DTF_{j\to i}\left(f\right)\right|}^{2}=0 & \forall i,j\in \left\{1,\ldots ,D\right\} \end{array} \end{array}$$ (11)

Rejecting $H_{0}$ at a specified significance level (typically α = 0.05) also required to reject non-statistically significant DTF values. Confidence intervals for the existing connections were estimated by determining the asymptotic distribution of DTF according to (Toppi et al., 2016). Only the thus identified statistically significant DTF values (ssDTF) were further analyzed in this study. Analogously to the DBI analysis, an index of directionality was finally obtained from the ssDTF estimates as follows:

$$\begin{array}{c} d_{i,j}\left(f\right)=\frac{ssDTF_{i\to j}\left(f\right)-ssDTF_{j\to i}\left(f\right)}{ssDTF_{i\to j}\left(f\right)+ssDTF_{j\to i}\left(f\right)} \end{array}$$ (12)

## Results

### Dynamical Bayesian Inference

**FIGURE 4:**
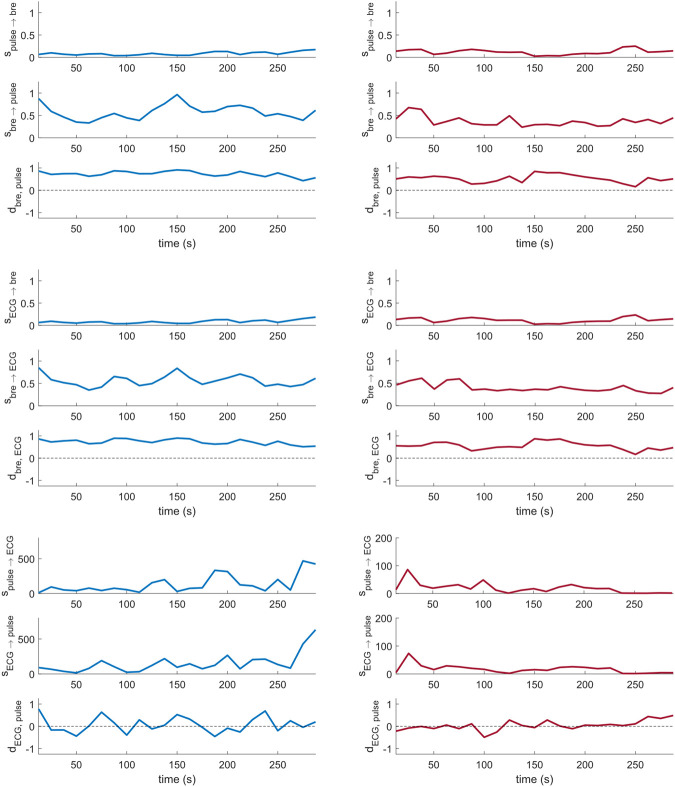
Cardio-respiratory directional coupling strength parameters estimated *via* DBI (Eqs 4, 5; two per panel), and respective directionality indices (Eq. 6; one per panel) obtained from a control subject (left) and a T1D patient (right).

**FIGURE 5:**
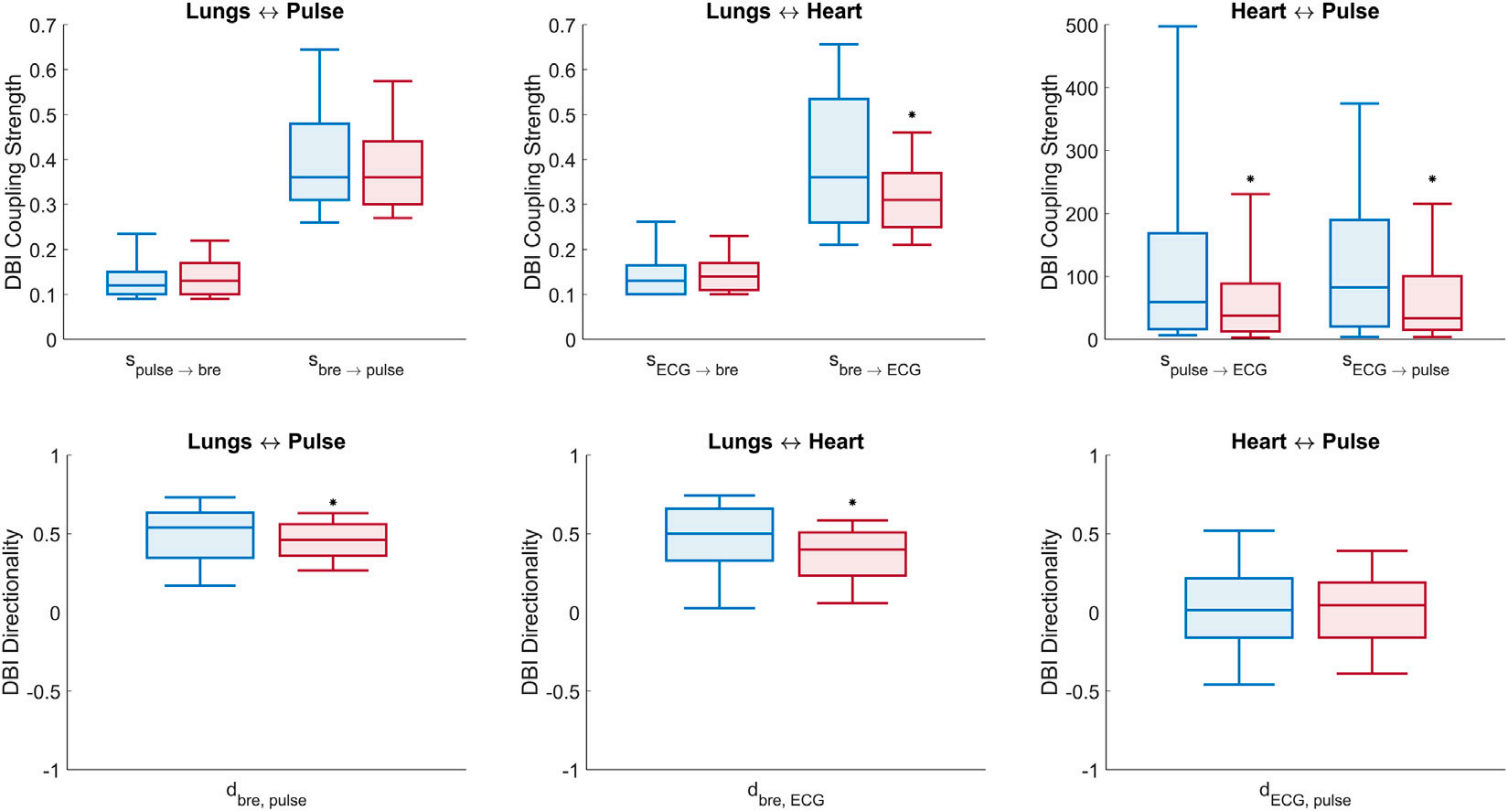
Box plots of DBI measures of connectivity (Eqs 4–6). Top panel: comparison of all (six) cardio-respiratory coupling parameters estimated with DBI for the control (blue) and T1D (red) groups. Bottom panel: comparison of the (three) directionality indices per pair interaction for the control (blue) and T1D (red) groups. Statistically significant decreases, identified by one-tailed Wilcoxon rank-sum tests, are denoted by (*) above the respective boxes.

Figure 4 shows sample coupling strength signals of the time-evolving pairwise interactions among peripheral pulse, respiratory and ECG signals, estimated using DBI in a control subject and a T1D patient. Directional coupling strength estimates below the corresponding median values of the surrogates reported in Table 3 (100 surrogate subjects, for a total of N_w_ = 2,300 windows), were rejected. Also, only those windows for which both directional coupling strengths per paired interaction were found to be statistically significant according to the above rule were further considered in the statistical analysis of the directionality index, $d_{i,j}$ . The overall results of the DBI analysis of the control and pathological groups including their statistical comparison (*p*-values) are shown in Figure 5 and summarized in Table 4. Statistically significant differences between the two groups were detected by means of one-tailed Wilcoxon rank-sum tests for independent samples.

**TABLE 3:** Median coupling strength values obtained from 100 inter-subject surrogates.

Interaction	DBI connectivity	Median surrogates (N_w_ = 2,300)
Lungs ↔ Pulse	$s_{pulse\to bre}$	0.08
	$s_{bre\to pulse}$	0.24
Lungs ↔ Heart	$s_{ECG\to bre}$	0.09
	$s_{bre\to ECG}$	0.19
Heart ↔ Pulse	$s_{pulse\to ECG}$	0.28
	$s_{ECG\to pulse}$	0.25

**TABLE 4:** Median of statistically significant (*p* < 0.05, vs. surrogates) DBI coupling strengths and directionality indices per interaction in controls and T1D patients. The statistical significance of the differences observed between the groups was estimated by one-tailed Wilcoxon rank-sum tests comparing the null hypothesis H_0_ (medians in the two groups are equal) to two alternative hypotheses: H_1a_, the median of controls being greater than that of T1D; and H_1b_, the median of T1D being greater than that of controls. The *p*-values for each of the two performed Wilcoxon tests (last two columns) refer to the probability of accepting hypothesis H_0_ to be true over the alternative hypotheses H_1a_ or H_1b_.

Interaction	DBI connectivity	Median (controls)	Median (T1D)	*p*-value H_1a_	*p*-value H_1b_
Lungs ↔ Pulse	$s_{pulse\to bre}$	0.120	0.134	0.949	0.051
	$s_{bre\to pulse}$	0.361	0.363	0.326	0.674
	$d_{bre,\;\;pulse}$	0.539	0.455	0.011^a^	0.990
Lungs ↔ Heart	$s_{ECG\to bre}$	0.127	0.140	0.887	0.113
	$s_{bre\to ECG}$	0.360	0.314	<0.001^a^	1.000
	$d_{bre,\;\;ECG}$	0.503	0.396	<0.001^a^	0.999
Heart ↔ Pulse	$s_{pulse\to ECG}$	59.21	37.51	<0.001^a^	0.999
	$s_{ECG\to pulse}$	82.86	33.44	<0.001^a^	1.000
	$d_{ECG,\;\;pulse}$	0.013	0.042	0.556	0.445

a Statistically significant at α = 0.05 level (one-tailed Wilcoxon rank-sum test).

Lungs–Heart interaction. Compared to controls, T1D patients exhibited a significant reduction in the directionality index $d_{bre,\;ECG}$ (*p* < 0.001), which reflects a lowered asymmetry of the cardio-respiratory interaction in the pathological group. This was due to a weakened influence of the breathing activity on the cardiac rhythm, as expressed by the statistically significant decrease in the directional coupling strength $s_{bre\to ECG}$ (*p* < 0.001; Table 4, row 4). Conversely, the directional coupling from the heart to the lungs was not significantly different between the two groups (Table 4, row 5).

Lungs–Pulse interaction. T1D patients also exhibited a significant decrease in the $d_{bre,pulse}$ index (*p* = 0.011), which indicates a higher symmetry of the interaction between the breathing activity and the cardiac oscillatory mode of the LDF signals. However, in this case, none of the corresponding directional coupling strengths was significantly different between the compared subjects (Table 4, rows 1 and 2).

Heart–Pulse interaction. With respect to the healthy group, T1D patients were characterized by significantly lowered directional coupling strengths, $s_{pulse\to ECG}$ (*p* < 0.001; Table 4, row 7) and $s_{ECG\to pulse}$ (*p* < 0.001; Table 4, row 8). However, no statistically significant difference emerged in the overall directionality of influence, as expressed by the $d_{ECG,pulse}$ index across the control and pathological groups (Table 4, row 9).

### Directed Transfer Function

**FIGURE 6:**
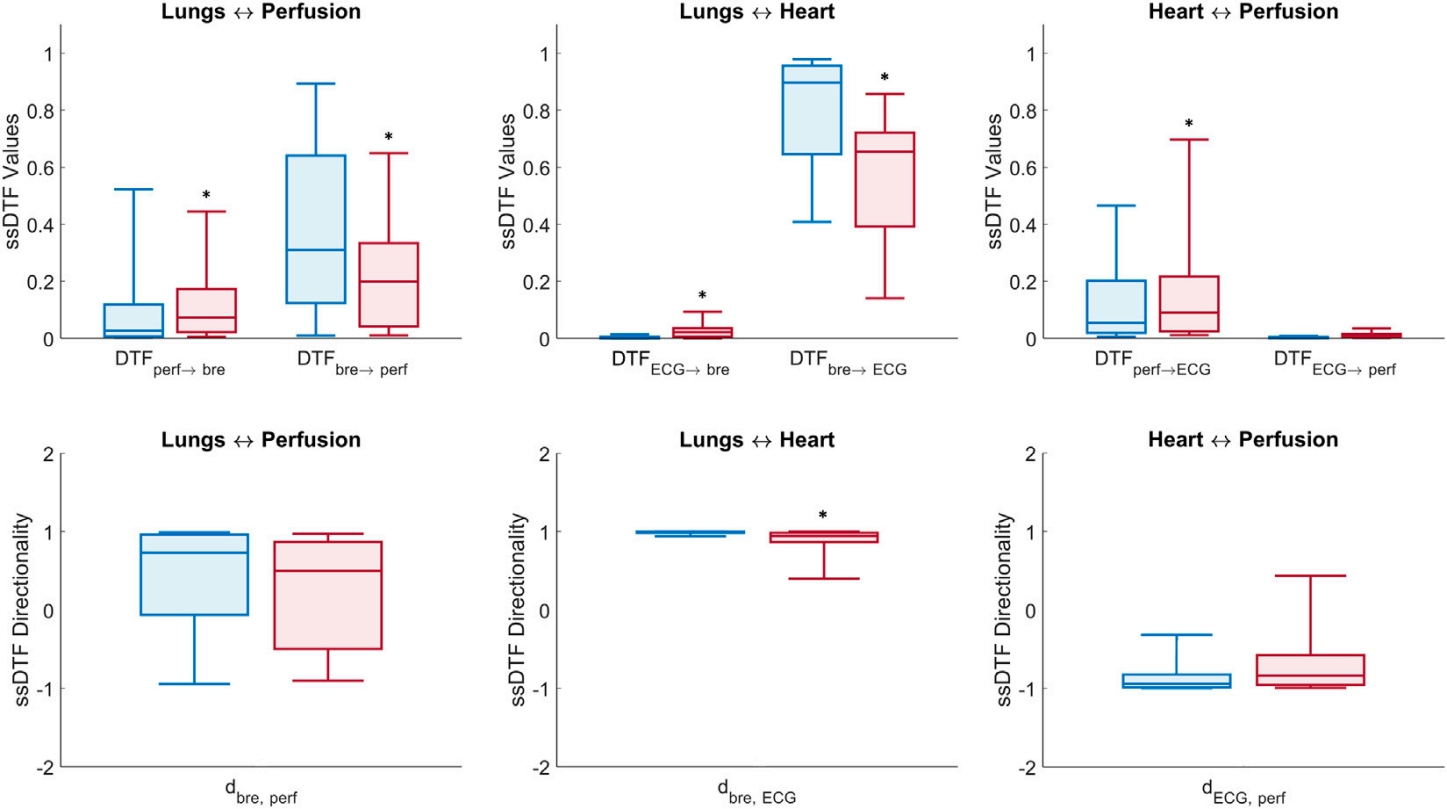
Box plots of DTF measures of connectivity in the (0.05, 2) Hz frequency band (Eqs 10, 12). Top panel: median ± first and third quartiles of ssDTF values across all directed interactions for control (blue) and T1D (red) groups. Bottom panel: directionality indices per pair interaction for the control (blue) and T1D (red) groups (*) above boxes denotes *p*-value < 0.05 estimated from non-parametric one-tailed Wilcoxon rank-sum tests comparing T1D and control groups.

The statistically significant DTF values (ssDTF) of directional connectivity estimated per interaction from MVAR modelling (six directional interactions between the three recorded signals) were aggregated over all windows (60-s non-overlapping data segments) and subjects within the same group (control or T1D) and averaged over the physiologically relevant frequency band (0.05, 2) Hz. The median and quartiles of the ssDTF values obtained from the control and T1D groups are shown in Figure 6. It is relevant to highlight that MVAR modelling evaluate signals across identical frequencies over the entire physiological range of interest, in contrast to DBI which is based on the extraction of the specific time-varying frequency component of the cardiac pulsatility, within an effectively tighter range. Therefore, in this section interactions involving LDF signals are denoted as “perfusion”, rather than “pulse”. From Figure 6, we make the following statistically significant observations about the assessed directional interactions: “Perfusion→Breathing”, “Perfusion→ECG” and “ECG→Breathing” connectivity strengths are elevated in T1D subjects compared to controls. Conversely, the “Breathing→ECG” interaction in T1D is lower than the controls’. It is also noteworthy that “Breathing→ECG” is significantly higher in connectivity than “ECG→Breathing” for both T1D and controls. Also, the “Perfusion→ECG” coupling is higher than “ECG→Perfusion” in both groups.

Statistically significant directional interactions (*p* < 0.05) between the network nodes for each pair of recorded signals are reported in Table 5 (columns 3 and 4) together with their directionality index $d_{i,j}$ . Inter-group comparisons were conducted via one-tailed Wilcoxon rank-sum statistical tests, whose *p*-values are also included in Table 5 (last two columns).

**TABLE 5:** Median of statistically significant (*p* < 0.05, vs. surrogates) DFT values (ssDTF) and directionality indices aggregated over subsequent time windows, and averaged over the frequency range (0.05, 2) Hz. The statistical significance of the differences observed between the control and T1D groups is also illustrated as in Table 4.

Interaction	DTF connectivity	Median (controls)	Median (T1D)	*p*-value H_1a_	*p*-value H_1b_
Lungs ↔ Perfusion	$ssDTF_{perf\to bre}$	0.027	0.074	0.982	0.019^a^
	$ssDTF_{bre\to perf}$	0.310	0.129	0.031^a^	0.970
	$d_{bre,\;perf}$	0.729	0.276	0.084	0.918
Lungs ↔ Heart	$ssDTF_{ECG\to bre}$	0.002	0.011	1.000	<0.001^a^
	$ssDTF_{bre\to ECG}$	0.897	0.634	<0.001^a^	1.000
	$d_{bre,\;ECG}$	0.992	0.954	<0.001^a^	1.000
Heart ↔ Perfusion	$ssDTF_{perf\to ECG}$	0.055	0.121	0.998	0.002^a^
	$ssDTF_{ECG\to perf}$	0.002	0.006	0.763	0.258
	$d_{ECG,\;\;perf}$	−0.940	−0.865	0.646	0.379

a Statistically significant at α = 0.05 level (one-tailed Wilcoxon rank-sum test).

Lungs–Heart interaction. In agreement with the results from the DBI method, DTF shows that T1D patients exhibit a statistically significant reduction (*p* < 0.001) in the directional coupling strength from the lungs to the heart, as well as in the directionality index $d_{bre,\;ECG}$ , compared to controls. The latter is due to a statistically significant increase (*p* < 0.001) in the directional strength $ssDTF_{ECG\to bre}$ observed in the pathological group, with a parallel significant decrease (*p* < 0.001) in $ssDTF_{bre\to ECG}$ (Table 5, rows 4–6).

Lungs–Perfusion interaction. Similarly to the DBI analysis, the DTF measures of connectivity showed a decrease in the $d_{bre,pulse}$ index of T1D patients compared to the one estimated from the control group (0.729 vs. 0.276), implying a lowered asymmetry of the interaction between breathing and LDF signals. However, this decrease was not as significant (*p* = 0.084) as the one estimated via DBI (*p* = 0.011). This outcome is due to the mixed results (decrease with *p* = 0.031, and increase with *p* = 0.019) related to the directional coupling strengths $ssDTF_{bre\to perf}$ and $ssDTF_{perf\to bre}$ , respectively (Table 5, rows 1 and 2).

Heart–Perfusion interaction. T1D patients exhibited increased connectivity compared to the controls in both directions, with the difference in the “Perfusion→ECG” coupling reaching statistical significance (*p* = 0.002) (Table 5, rows 7 and 8). The above trends contributed to a diminished absolute value of the directionality index $d_{ECG,perf}$ in the pathological group, which implies a more balanced interaction with respect to controls (Table 5, row 9).

Finally, within each group, a few interesting features were also observed from the DTF results in relation to the difference in the strength of the directional couplings per interaction. In particular, in both control subjects and T1D patients, the median coupling strengths from the lungs to the heart and from the lungs to the microcirculation were considerably higher than in the opposite direction (the same outcome of the DBI analysis). However, the inter-group differences in the directional strengths between heart and microcirculation were contradictory with respect to the DBI analysis, which associated a higher level of bidirectional connectivity to the control group (Table 5, columns 3 and 4).

## Discussion

Towards the goal of developing reliable and non-invasive biomarkers for T1D, we employed both nonlinear (bivariate) and linear (multivariate) measures to assess possible impairments in the coupling strength and directionality of influence between three representative nodes of the cardiovascular and respiratory systems (heart, lungs, microcirculation) in patients diagnosed with T1D compared to control subjects. The two adopted methods can capture equivalent or different features in the communication between the nodes of a physiological network because of their different capabilities, that is: linearity (DTF) vs. nonlinearity (DBI) in the data; multivariate (DTF) vs. bivariate (DBI) data analysis; measure of connectivity between signals at the same frequency (DTF) vs. different frequencies (DBI). Employing these two techniques, we did identify impairments (by both or one of the approaches) in the functional directional interactions between heart, lungs, and microcirculation in T1D patients. In detail, an impairment was defined as a statistically significant difference (*p* < 0.05) in the directional coupling strengths between the respective nodes, compared to the homologous estimate obtained from the control group (i.e., rejection of the null hypothesis H_0_).

Regarding the functional interactions between heart and lungs, DBI, the nonlinear framework, revealed a significantly reduced (*p* < 0.001) influence of the respiratory activity on the phase of the cardiac rhythm in the T1D group. A similar, statistically significant (*p* < 0.001) finding also emerged from the linear network analysis, using DTF. Moreover, the imbalance in the two communication channels from the lungs to the heart and vice versa, as captured by the directionality index, was also highly significantly different in both methods (*p* < 0.001). It is well known that the phase of the respiratory activity directly influences the action of the heart pump, as breathing-related changes in the intrathoracic volume alter the cardiac pre-load, thus affecting cardiac filling, post-load and other circulatory variables. Furthermore, respiration gates the timing of autonomic motoneuron firing (Eckberg, 2003), thus modulating the peripheral autonomic nervous system’s outflow to the heart, an indirect cardio-respiratory coupling occurring via neuronal control (Iatsenko et al., 2013; Kralemann et al., 2013). Therefore, our finding of a reduced driving relationship of the lungs to the heart in T1D patients could be related to autonomic neuropathy, vascular degeneration or lung tissue stiffening, common co-morbidities associated with diabetes mellitus (Klein et al., 2010).

An analogous decrease of the influence of respiration on the microvascular perfusion in the T1D group compared to controls was observed by DTF analysis (*p* = 0.031) but could not be verified by DBI (*p* > 0.05). However, like for the lungs-heart interaction, the imbalance in the directional coupling strengths between lungs and microcirculation, as reflected by the directionality index, was significantly less (*p* = 0.011) in T1D than in controls as shown by DBI as well as by DTF, though without reaching a statistical significance level (*p* = 0.084). Also, regarding the DBI analysis of phase interactions, it is notable that control subjects exhibited similar statistics with respect to the evaluation of breathing and ECG signals. This result would be in line with previous findings by Jamšek and Stefanovska on the coupling information among cardiac and respiratory processes which propagates to the distal microvascular beds (Jamšek and Stefanovska, 2007), and can be characterized through the analysis of LDF signals recorded non-invasively from the skin.

In T1D subjects, the DBI analysis highlighted a significantly decreased communication in both directions between the ECG and the microvascular pulse signal extracted from LDF signals. This finding, however, could not be validated by DTF too. It is noteworthy that these directional interactions were associated with significantly higher coupling strength values (Table 4). This could be due to the way DBI evaluates causal relationships and what it can capture. In this case, DBI basically assesses the phase coupling between ECG and pulse signals that, although recorded at different anatomical locations, originate from the same source, representing the electrical and mechanical activities of the heart, respectively (Kralemann et al., 2013).

Finally, the estimated directional couplings from the lungs to the heart and microvasculature, via either the DBI or DTF methods, were considerably higher than the ones from the heart and vasculature towards the lungs, in both control subjects and T1D patients. Since this outcome was common in both groups, it cannot be used as a biomarker for T1D. However, it agrees with the findings of Palus and Stefanovska (2003), which have shown that the respiratory process drives the heart activity at all breathing frequencies, whether paced or spontaneous, and may shed more light on the involved physiological mechanisms *en route* to a better understanding of the cardio-respiratory system.

A potential limitation of this study is the availability and analysis of signals from only a small number of nodes (lungs, heart, microcirculation) in the network under investigation. Both DTF and DBI measure the global (direct and indirect) interactions between two nodes A and B, the indirect interactions from A to B or from B to A occurring through other node(s) C that we may not have access to in the network (Kamiński et al., 2001; Baccalá et al., 2016). In this regard, it is established that each respiratory cycle is tightly controlled by four separate control centers in the pons and medulla (Smith et al., 1991; Hilaire and Pásaro, 2003; Dampney, 2017), which cannot operate without central intervention from the brain, and direct feedback from the heart. Furthermore, central autonomic neural control has a well-known role in the low- and high-frequency variability of the heart rate (Shaffer and Ginsberg, 2017). Thus, ignoring the brain (EEG) and investigating this complex neuro-cardio-respiratory network from only three nodes (lungs, heart, microcirculation) could have skewed the level of the estimated bivariate interactions in both T1D and control groups. However, the comparative statistical analysis of each measure across the two groups may take care of this skewness if it were in the same direction in both groups, per interaction.

In summary, we found that in both control and T1D subjects, breathing had greater influence on the heart and peripheral microvascular perfusion, compared to the opposite directional couplings and that, by both the employed methods of connectivity analysis, the causal influence of the respiratory activity on the heart was significantly decreased (*p* < 0.05) in T1D patients compared to the control group. These preliminary results can be linked to established comorbidities of T1D and, although obtained from a limited number of subjects, provide a strong indication for the usefulness of a network-based multi-modal analysis for the development of biomarkers from short-duration data, and for monitoring the disease and T1D-related complications over time, as well as its potential in the exploration of the pathophysiological mechanisms that underlie this devastating and very widespread disease.

## Data Availability

The raw data supporting the conclusion of this article will be made available by the authors, without undue reservation.
